# Integrated Sequence-Structure Motifs Suffice to Identify microRNA Precursors

**DOI:** 10.1371/journal.pone.0032797

**Published:** 2012-03-15

**Authors:** Xiuqin Liu, Shunmin He, Geir Skogerbø, Fuzhou Gong, Runsheng Chen

**Affiliations:** 1 School of Mathematics and Physics, University of Science and Technology Beijing, Beijing, PR China; 2 Institute of Zoology, Chinese Academy of Sciences, Beijing, PR China; 3 National Laboratory of Biomacromolecules, Institute of Biophysics, Chinese Academy of Sciences, Beijing, PR China; 4 The Key Laboratory of Random Complex Structures and Data, Chinese Academy of Sciences, Beijing, PR China; 5 National Center for Mathematics and Interdisciplinary Sciences, Chinese Academy of Sciences, Beijing, PR China; 6 Institute of Applied Mathematics, Academy of Mathematics and System Sciences, Chinese Academy of Sciences, Beijing, PR China; University of Edinburgh, United Kingdom

## Abstract

**Background:**

Upwards of 1200 miRNA loci have hitherto been annotated in the human genome. The specific features defining a miRNA precursor and deciding its recognition and subsequent processing are not yet exhaustively described and miRNA loci can thus not be computationally identified with sufficient confidence.

**Results:**

We rendered pre-miRNA and non-pre-miRNA hairpins as strings of integrated sequence-structure information, and used the software Teiresias to identify sequence-structure motifs (ss-motifs) of variable length in these data sets. Using only ss-motifs as features in a Support Vector Machine (SVM) algorithm for pre-miRNA identification achieved 99.2% specificity and 97.6% sensitivity on a human test data set, which is comparable to previously published algorithms employing combinations of sequence-structure and additional features. Further analysis of the ss-motif information contents revealed strongly significant deviations from those of the respective training sets, revealing important potential clues as to how the sequence and structural information of RNA hairpins are utilized by the miRNA processing apparatus.

**Conclusion:**

Integrated sequence-structure motifs of variable length apparently capture nearly all information required to distinguish miRNA precursors from other stem-loop structures.

## Introduction

More than 1200 miRNAs have been identified in humans [1]. The characteristics defining a miRNA locus are not yet known in all detail, and computational methods for identification and annotation of new miRNAs still need improvement. Machine learning algorithms represent a set of regularly and widely used methods for classification of various types of information, and a number of research groups have used machine learning to predict new miRNA loci [2]–[13]. It is evident from comparison of these methods that the features used for pre-miRNA detection can heavily influence the performance of a method (see also Table 1 in Jiang *et al.* [13]). However, the use of empirically derived miRNA characteristics in computational analysis is not straightforward, and features commonly employed for computational miRNA detection or identification lead to substantial differences in performance.

**Table 1:** Comparison between Mirident and previously published software/algorithms.

	ACC(%)	SP(%)	SE(%)	AUC(%)	Ref
Mirident	98.39	99.19	97.58	99	
3SVM^1^	83.87	89.52	78.23	n.a.	[2]
Mir-albra(Th^2^ = 0)	80.242	1	60.48	n.a.	[18]
Mir-albra(Th^2^ = −1)	89.5	95.97	83.65		
Mir-albra(Th^2^ = −2)	81.45	69.35	93.55		
PmirP	89.1	95.97	82.26	n.a.	[12]

1 Original training data.

2 “Th” indicates “Threshold”.

All the models were tested on the same data set of 124 pre-miRNAs and 124 non-pre-miRNA hairpins.

miRNAs are processed from longer precursor transcripts (pri- and pre-miRNAs), and it is the processing apparatus which ultimately decides whether an RNA hairpin structure shall constitute a miRNA locus or not. Recent analyses show that the pri-miRNA structure is recognized in a co-operative manner by the Microprocessor component DGCR8 [14]. DGCR8 binds the pri-miRNA stem-loop structure as a trimer, resulting in a large interacting surface which probably allows for numerous and variable points of interaction. Simultaneous employment of sequence and structure information has been shown to yield higher predictability of miRNA loci than expected from their additive influence effects [15]. Inclusion of local contiguous structure-sequence information for distinguishing pre-miRNA loci from other potential hairpin structures was first reported by Xue *et al.* [2], and various combinations of sequence and secondary structure features have also been applied by other studies [3], [5]. Recently, Zhao *et al.* [12] used a support vector machine (SVM) with short sequence-structure features (in combination with additional information) to discriminate actual pre-miRNAs from other potential hairpin structures, achieving 94.9% sensitivity and 98.4% specificity on a human test set.

Combinations of sequence-structure information have been shown to lead to progress in pre-miRNA prediction [2], [12], [13], [16], however previously published methods have only applied sequence-structure features of fixed size. The present study integrated sequence and secondary structure characteristics into a single information string of variable length, and may thus better capture the real features of pre-miRNAs and other RNA hairpins. We utilized this idea to carry out exhaustive searches for all possible sequence-structure motifs (ss-motifs) on potential RNA hairpin structures. Applied within a loosely defined sequence-structure space (*e.g.*, predicted stem-loop structures) a machine learning algorithm should be able to predict precursor miRNAs based on the identified sequence-structure motifs. To test this hypothesis we developed an SVM algorithm (Mirident), which, when employing the 1300 most informative ss-motifs, was able to predict miRNA loci in the human genome with higher specificity and sensitivity than any other previously published computational tool.

## Results and Discussion

### The sequence-structure motif

The functionality of an RNA molecule is predominantly determined by its primary nucleotides sequence and the intra-molecular interactions (hydrogen-bonding) deciding its secondary or 3-dimensional structure. These two modes of molecular information have conventionally been represented by a string of letters (*e.g.*, UUCCCAAAGUUGAGAA) denoting the chemical composition of a 16 nucleotides long RNA molecule, and a string of brackets and dots (*e.g.*, “(((.((….)).)))”) denoting the intra-molecular interactions forming the basis for its secondary structure. In a molecular and functional context the combination of both aspects are probably of high importance. To be able to identify molecular features which combine sequence and structural information, we therefore integrated both sequence and secondary structure into a common information string (ss-string). Replacing the structure symbols “(”, “.” and “)” by “L”, “D” and “R”, respectively, and adding these as subscripts to each respective nucleotide notation, the chemical composition and the intra-molecular structural of the above RNA molecule can be represented by a single information string, *i.e.*, U_L_U_L_C_L_C_D_C_L_A_L_A_D_A_D_G_D_U_D_U_R_G_R_A_D_G_R_A_R_A_R_ (“N_S_” denoting “any nucleotide, any intra-molecular interaction”). From these ss-strings we extracted frequently occurring motifs (ss-motifs) of varying length (see Materials and Methods) which were subsequently used to distinguish pre-miRNAs from other stem-loop structures.

### Motif extraction and evaluation

**Figure 1:**
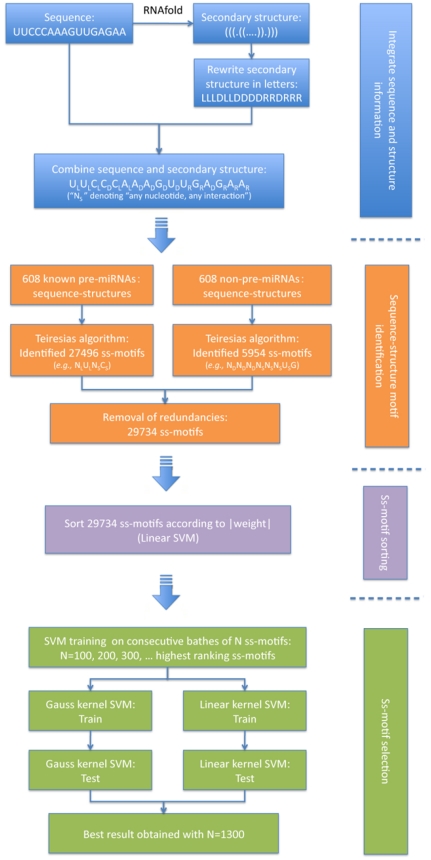
The Mirident pipeline.

To test the efficacy of the ss-motifs in distinguishing miRNA precursors from RNA stem-loop structures not encoding miRNAs, we developed an SVM-based classifier for prediction of pre-miRNAs (Mirident, Figure 1). The software Teiresias [17] was used to search for ss-motifs in 608 verified pre-miRNA hairpins (positives), and 608 non-pre-miRNA hairpins extracted from coding regions of the genome (negatives). From the 608 positive and 608 negative hairpins, 27496 and 5954 ss-motifs were extracted, respectively, of which remained a total of 29734 ss-motifs when redundancies were removed. Computing the frequency of each motif created a 29734×1216 feature matrix which was used to construct a classifier. As the high number of ss-motifs very likely contained redundant information, we used a linear SVM algorithm to estimate a weight for each ss-motif (see Materials and Methods), according to its contribution to distinguishing positives from negatives. The ss-motifs were subsequently ranked in descending order according to their weights (Table S1).

### Mirident efficiently identifies miRNA precursors

**Figure 2:**
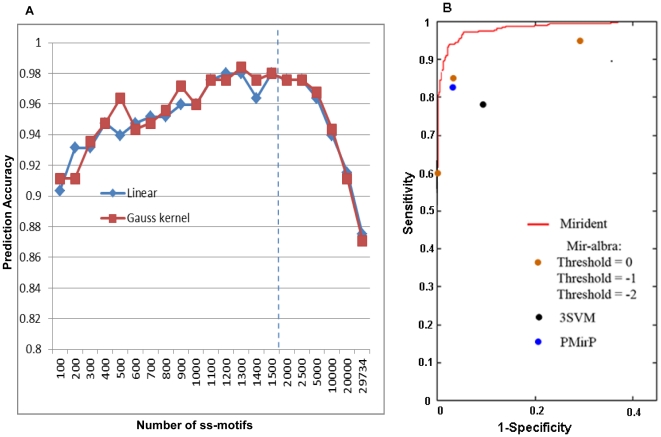
Mirident performance. A. Effect of increasing number of ss-motifs on miRNA prediction accurracy. Note: The X-axis is discontinuous above 1500 motifs (dashed line). B. The ROC curve of Mirident (red line) trained with 1300 ss-motifs. The Area Under Curve (AUC) is 0.99. Results for other methods are shown for comparison.

By successively selecting the N ss-motifs with the highest weights (N = 100, 200, 300,…‥, 29734) for training of the linear classifier, and subsequently employing it to predict pre-miRNA hairpins (see Materials and Methods), we obtained a measure of the prediction accuracy for each increment in the number of ss-motifs (Figure 2A and Table S2). From Figure 2A it can be seen that the prediction accuracy increases with increasing number of ss-motifs until approximately 1300 ss-motifs have been included, at which the prediction accuracy reaches its maximum value (98.39%), corresponding to specificity and sensitivity values of 99.20% and 97.58%, respectively. Further inclusion of ss-motifs led to a decline in prediction accuracy. As the above results were obtained with a linear kernel SVM, we repeated the procedure using a Gaussian kernel SVM in order to further validate the result. As can be seen from Figure 2A, the results obtained with the Gaussian kernel SVM deviated little from those obtained with the linear kernel SVM.

The results obtained with Mirident is comparable to those of previously published computational methods for pre-miRNA prediction, *e.g.* miR-abela [18] and the 3SVM classifier [2] (see Table 1). A more detailed comparison was made between Mirident and the PMirP method [12], for which very high sensitivity (98.4%) and specificity (94.9%) was reported when applied on human pre-miRNA and hairpin data. PMirP [12] is based on sequence-structure triplets, but also includes minimum free energy (MFE) and overall base-pairing data. When applied to the human test set used in the present study, PMirP achieved a sensitivity of 82.26% and a specificity of 95.97%, which falls somewhat behind the performance obtained with Mirident. Figure 2B delineates the ROC curve for Mirident, giving an Area Under the Curve (AUC) value of 0.99, which further emphasize the potential of ss-motifs of pre-miRNA prediction.

**Figure 3:**
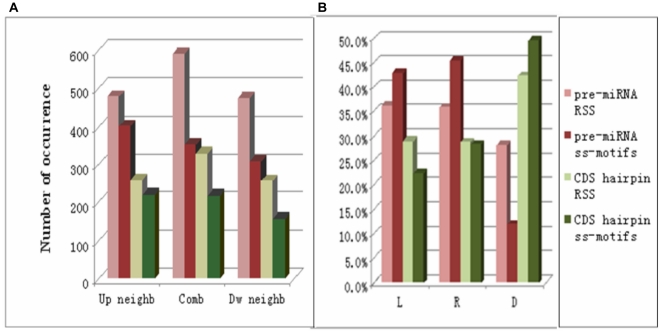
Sequence-structure motif characteristics. Light hues (pink, light green) indicates the positive and negative randomly selected sequences (RSS). Darker hues (red, green) indicates the actual ss-motifs derived from the positive (pre-miRNA) and negative (CDS hairpin) training sets. A. Combinations of nucleotide and structural information in the ss-motifs. The figure shows occurrence of structural information relative to positions with specific nucleotide information. “Comb” denotes occurrences of specific nucleotide and structural information combined at the same position (*e.g.*, “A_L_”, “C_D_”, etc). “Up neighb” and “Dw neighb” denote occurrences of specific nucleotide notation combined with specific structural notations at the nearest upstream or downstream neighbouring position (*e.g.*, “N_L_A_S_” etc, and “A_S_N_L_” etc), respectively. B. Distribution of “L”, “R” and “D” denotes “left”, “right” and absence of (notation of) intramolecular interactions, respectively.

The results obtained with Mirident suggest that ss-motifs efficiently capture the essential characteristics of miRNA precursors. The difference between Mirident and previously published methods employing integrated sequence-structure information [2], [12] may reside in the more flexible manner in which Mirident harvests this information by extracting sequence-structure motifs of undefined length, and the larger number of informative features this methodology achieves. The observations reported below that integrated nucleotide and structural information is not confined to the nearest nucleotide (Figure 3A) further suggests that longer ss-motifs may have an edge over sequence-structure triplets. Taken together, the results obtained in the tests above would suggest that a substantial amount of the information needed to distinguish miRNA precursors from non-pre-miRNA hairpins can be contained in a set of ss-motif.

**Figure 4:**
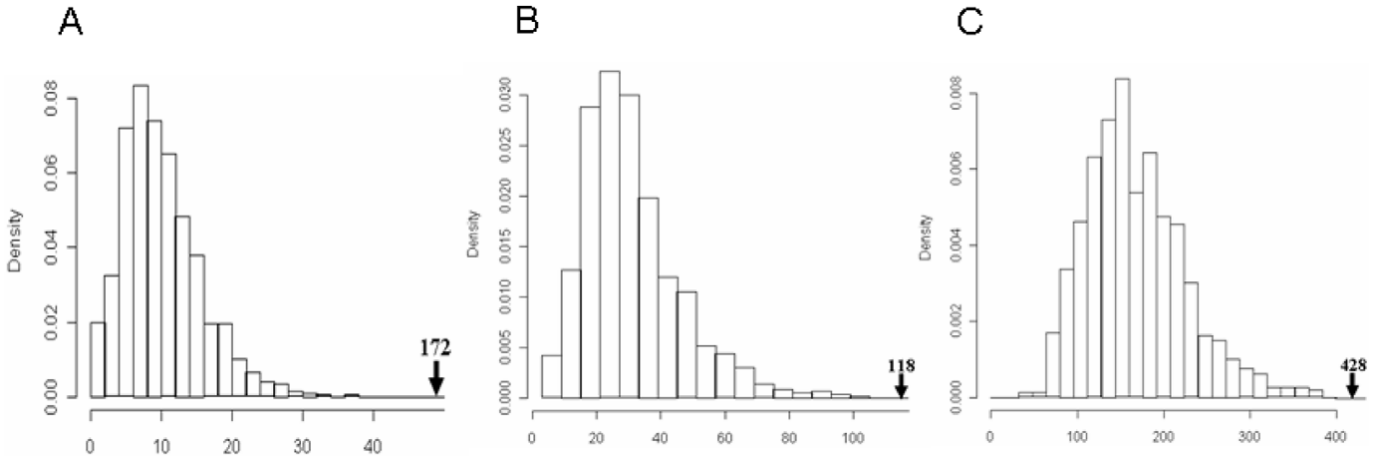
Increased ss-motif similiarity within miRNA families. The panels show the average number of common ss-motif among members of each miRNA family (arrow), compared to a distribution of average pre-miRNAs (repeated 1000 times). A. The miRNA gene family mir-515 (26 members; 172 common ss-motifs). B. The miRNA gene family mir-154 (17 members; 118 common ss-motifs). C. The miRNA gene family let-7 (8 members; 428 common ss-motifs).

An additional question concerns how the sequence-structure information is distributed within miRNA families. While mature miRNA sequences are generally very similar within miRNA families, the sequences of miRNA precursors vary considerably. To investigate this, we compared the number of ss-motifs common to all members of a miRNA family to that of the same number randomly selected miRNA precursors (the random selections being repeated 1000 times). As shown in Figure 4, miRNA family members had significantly (p<0.001) more ss-motifs in common than had randomly selected miRNA precursors, which may bias the Mirident performance if members of the same miRNA family occur in both test and training set. On the other hand, only 29 of the pre-miRNAs in the test set (altogether 124 pre-miRNAs) had a family member in the training set, and even if these 29 pre-miRNAs were excluded, the detection rate for the rest of the set was 94.7% (90/95). We nonetheless repeated the entire Mirident procedure after filtering for precursors with sequence identity above 80% or 70%, and reached prediction accuracy values of 97.4% and 96.9% (Table S3), respectively (see Supporting Information S1 for details). Thus, while not being able to entirely exclude a “family” effect on the prediction performance, we do not think this influence can be strong.

### Mirident identifies novel and non-human pre-miRNAs

Although the 1300 ss-motifs employed by Mirident are derived from human hairpin structures, these motifs may be representative of miRNA precursors of most organisms. We therefore applied Mirident to the 5034 single loop non-human pre-miRNA sequences in the miRBase version 11.0 [1], of which the algorithm was able to distinguish 93.8% (Table 2). Between the miRBase versions 11.0 and 17.0, 9372 single-loop pre-miRNA hairpins were entered into the database, of which 88% were identified (Table 2). When applied to specifically to all mouse and rat pre-miRNAs in miRBase version 17, Mirident identified 88.2% and 93.0% of these, respectively. Similarly, when applied to the pre-miRNAs of four different viruses, Mirident identified from 92.3% to 100% of these (Table 2).

**Table 2:** Mirident prediction accuracy on non-human and novel pre-miRNA data sets.

Species	Number of pre-miRNAs	Accuracy (%)
All non-human pre-miRNAs in mirBase V11.0	5034	93.8
Recent pre-miRNAs (all species; miRBase v.12–17)	9372	88.0
Mouse (Mus musculus),miRBase v17	720	88.2
Rat (Rattus norvegicus), miRBase v17	408	93.0
EBV	25	100
HCMV	11	100
MGHV68	15	93.0
KSHV	13	92.3

### The ss-motif information content differ from that of the respective training sets

The distribution of notations in the 1300 ss-motifs might give clues to the nature of informational content in the miRNA precursor. The average ss-motif was 6.3 nucleotides long (Table S1), and the 1300 ss-motifs contained 6431 specific notations (“N” and “S” excluded), with a substantial bias towards structural information (72.9% of all notations) (Table S4). More motifs were extracted from the positive (pre-miRNA; 941 ss-motifs) than from the negative (553 ss-motifs) training set (Table S1). With respect to nucleotide content, the number of U notations in the pre-miRNA ss-motifs was greatly enriched (p<10^−100^) above the corresponding training set, whereas the number of C and A notations are greatly reduced (p<3×10^−12^). In the CDS hairpin ss-motifs, G notations are significantly enriched (p = 1.48×10^−12^) and U notations significantly (p = 3.57×10^−5^) depleted relative to the CDS training set (Figure S1 and Table S4).

To further analyze the information content of the ss-motifs, we compared the statistics of the ss-motifs to randomly selected sequences (RSSs) from the respective training sets (see Supporting Information S1). The structural notations (*i.e.*, “L”, “R” and “D”) of the pre-miRNA ss-motifs were significantly enriched for both left (“L”) and right (“R”; p = 3.51×10^−6^ and p = 7.81×10^−11^, respectively) notations relative to the pre-miRNA training set, whereas the CDS hairpin ss-motifs were significantly enriched in specific “D” notations (p = 2.73×10^−7^) relative to the negative training set (Figure 3B and Table S4). Thus, the differences between the training sets were accentuated in the two ss-motifs sets. Tentatively, the data suggest that the presence of specific intra-molecular interactions (*i.e.*, L and R) may have a defining value for both miRNA and non-miRNA precursors. Contrarily, while information on absence of intra-molecular interactions at specific nucleotide position (or combinations of positions) has little positive value for defining a miRNA precursor as such, this type of information may have a strong defining value with respect to non-miRNA precursors.

Given that the input information in the sequence-structure strings is composed of integrated nucleotide and intra-molecular information (*e.g.*, “A_L_”, “C_D_”, *etc*), it might be expected that the output information (in the ss-motifs) would take the same form. However, significantly fewer positions in both the pre-miRNA and CDS hairpin ss-motifs contained combined nucleotide and structural notations (*e.g.*, A_L_) than in randomly selected sequences from their respective training set sets (p = 4.20×10^−52^ and p = 2.11×10^−23^, respectively; Figure 3A). Also, structural notations for neighboring nucleotides were significantly less frequent than expected (see Figure S2), suggesting that informative nucleotide and structural notations that frequently are located more than one nucleotide residue apart, which would imply that the miRNA processing apparatus utilizes combinations of well-spaced sequence and structural information in the recognition or rejection of specific hairpin. This may also go some way to explain the relatively lower efficacy of sequence-structure triplets [2], [12] in predicting miRNA hairpins. (Further details on the ss-motif information content are found in Supporting Information S1).

### ss-motif position and distribution

**Figure 5:**
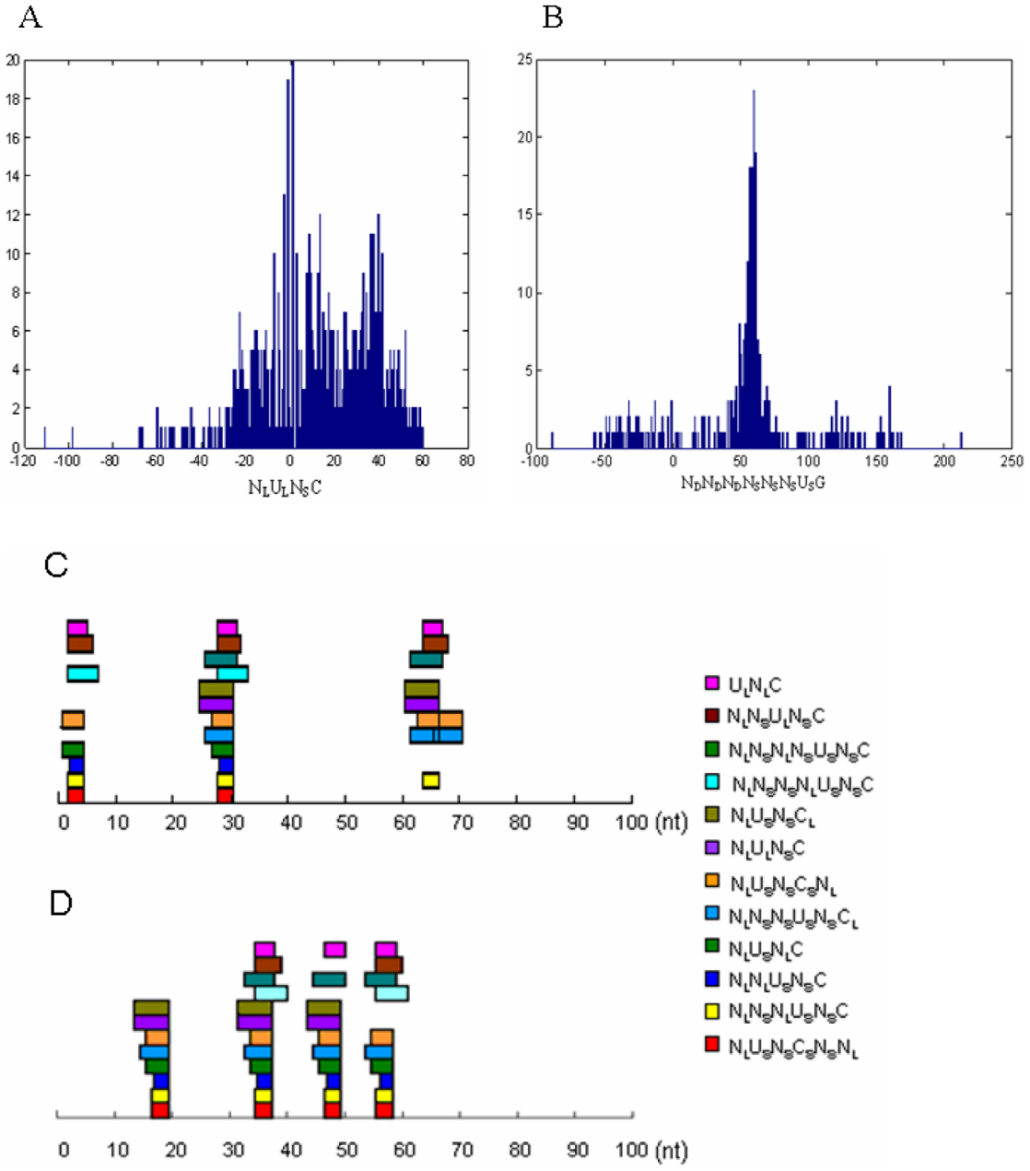
ss-motif positions. A. ss-motif N_L_U_L_N_S_C_S_ is frequently found at the 5′end of the pre-miRNA sequence. B. ss-motif N_D_N_D_N_D_N_S_N_S_N_S_U_S_G_S_ is concentrated at position 50 in the loop. C&D. Multiple position of similar ss-motifs along the pre-miRNA left strand of has-miR-1302-4 (C) and has-miR-548i-1 (D). It should be noted that the figure shows ss-motif positions in extended pre-miRNA sequences (see Materials and Methods).

In order to see whether the individual ss-motifs occur at specific positions, along the pre-miRNA stem-loop structure we plotted the positions of the ss-motifs along the pre-miRNA sequence (see Supporting Information S1 for details). Overall, very few ss-motifs were located at any specific position (Figure 5A), and the same (or very similar) ss-motifs commonly occurs at several positions along the stem part of the hairpins (Figure 5C&D; further analysis of motif correlations is found in Supporting Information S1). The relatively few ss-motifs that occupied very specific locations were often located in the loop of non-pre-miRNA hairpins (Figure 5B). Further analysis also suggested that ss-motifs with a G residue but few or none structural notations were frequent among ss-motifs from the non-pre-miRNA set; thus, the sequence-structure information in the loop may be more important for rejection of non-miRNA hairpins by the miRNA processing apparatus than for recognition of actual miRNA precursors. This observation is in agreement with experimental data showing that the loop is not absolutely required for processing of a pri-miRNA (*e.g.*, has-miR-16) by the Drosha-DGCR8 complex in vitro [19]. Thus, the presence of a loop may not be an absolute requirement for the recognition and/or processing for most miRNA precursors, the loop may still contain information that is inhibitory to its recognition or processing. On the other hand, it has been shown that human miRNA loops contain conserved binding sites for various proteins that either promotes or inhibits miRNA precursor processing [20]–[24], but the fraction of miRNAs with conserved loop sequences (around 14% [20]) may have been too small to make a strong mark on the overall ss-motif composition.

If the processing of miRNA precursors into mature miRNAs by the Microprocessor and Dicer complexes are considered as enzymatic reactions in which cooperative interactions between substrate and enzyme leads to an orientation and arrangement of both molecules which elicit the enzymatic reaction [25], it is tempting to see the ss-motifs as a nearly complete catalogue of the pri-/pre-miRNA surface features that enable their recognition and processing. It is a reasonable assumption that the specific interactions between the precursor miRNA and the enzyme protein surface will occur on a number of compatible “micro-domains” of the surfaces of the respective molecules. The interacting micro-domains on the precursor miRNA will be specified by a combination of spatial and electro-chemical properties, which in turn are determined mainly by the primary sequence and its intra-molecular interactions of the molecule. In comparison to the enzyme kinetics of small molecules, where most interactions between substrate and enzyme must occur in or in the immediate vicinity of the active site, both the precursor miRNA and the processing complexes are relatively large molecules with extended molecular surfaces, enabling a large number of possible interacting micro-domains. On the other hand, although a large number of potential interactions may exist, a limited number of cooperative interactions may in any specific case be sufficient to achieve the required coordination of substrate and enzyme that elicits the enzymatic reaction. The large number of informative and non-correlated ss-motifs identified in this study suggests that a hypothetical miRNA precursor may interact with the processing enzymes in a large number of different ways, the only necessary and sufficient criterion being that the sum of the interactions must achieve an orientation and arrangement of substrate and enzyme which elicits the enzymatic reaction. An actual miRNA precursor may, on the other hand, only realize a few of these numerous potential combinations of interactions in order to produce a mature miRNA, and an exhaustive catalogue of criteria defining a miRNA precursor may therefore be difficult to obtain by empirical methods.

## Materials and Methods

### Data

#### Human pre-miRNA sequences

miRNA precursor sequences from the miRNA database miRBase version 11.0 [26] were filtered for multi-loop structures and redundancies (*i.e.*, sequence identity >90%; see Supporting Information S1 for details) to yield a final set of 608 human miRNA precursor sequences. Although these sequences will in a number of cases contain some flanking sequence beyond the actual pre-miRNA sequence, we will for simplicity refer to this sequence set as the “pre-miRNA set”.

#### Human non-pre-miRNA stem-loop structures

Predicted stem-loop structures from coding domains (CDSs) of human genes (UCSC [27], [28]) were used to generate a set of non-pre-miRNA sequences according to five specific criteria (see Supporting Information S1 for details). These sequences have never been reported to generate mature miRNAs.

*Training sets*. Training sets of 484 pre-miRNA sequences (positive training set) or 484 non-pre-miRNA sequences (negative training set) were obtained by random selection among the 608 sequences in the pre-miRNA and non-pre-miRNA sequence sets, respectively.

*Test sets*. Four types of test sets were used. The first type included 124 pre-miRNA and 124 non-pre-miRNA sequences from the above human pre-miRNA and non-pre-miRNA sets after removal of the sequences used for the training sets. The second type included 5034 single loop hairpin structure of non-human pre-miRNA sequences from miRBase (version 11.0) [26]. The third type included 9372 pre-miRNA sequences predicted to form single loop hairpin structures that were entered into the miRBase from version 12.0 to version 17.0. The fourth type is rat, mouse and four viruses (Epstein Barr Virus, Human cytomegalovirus, Mouse gammaherpesvirus 68, Kaposi sarcoma-associated herpesvirus) down-loaded from miRBase (version 17.0).

### ss-motif extraction

The software Teiresias [17] was used to separately search for frequently occurring sequence-structure motifs (ss-motifs) of variable length in the two sets of 608 pre-miRNA and 608 non-pre-miRNA sequences, respectively. The options used were “Exact discovery”, “Seq Version” and “Accept all characters”. The parameters used were L = 4, W = 12, and K = 457,which briefly implies that any motif of length W = 12 positions contains at least L = 4 defined nucleotide or structural notations and occurs in at least K = 457 different sequences, will be retained. For instance, if the motif U_S_N_S_N_S_N_S_N_L_A_L_N_L_U_S_N_S_N_S_N_L_′ is found in a pre-miRNA ss-string, this can be subdivided into the two separate motifs U_S_N_S_N_S_N_S_N_L_A_L_ and A_L_N_L_U_S_N_S_N_S_N_L_, each of length W = 12, and both containing at least L = 4 defined nucleotide or structural notations.

### Assigning weights to the ss-motifs

We used an SVM with linear kernels to assign a weight 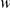 to each ss-motif. The method generally followed that of Brank *et al.* [29] for normal-based feature selection. Briefly, assuming n support vectors and a total of k ss-motifs, the matrix of the “model file” is given as: 
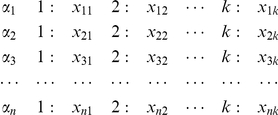



The weight 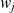 is then calculated as: 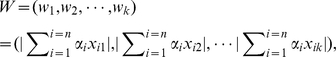
Using 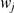 to denote the weight of the j^th^ 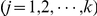 ss-motif, the motifs were sorted according to descending weight (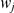).

### SVM for training and prediction

A Support Vector Machine (SVM) procedure was adopted to classify pre-miRNAs versus non-pre-miRNA hairpins using the 968 sequences in the training sets as input. After sorting the motifs by “
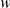
” (the weight of the ss-motifs) obtained with the linear kernel SVM model, we sequentially introduced batches of 100 ss-motifs from the top of the sorting list until an optimal performance (ACC = 98.39%) was reached at 1300 ss-motifs, where after the accuracy decrease with increasing number of ss-motifs. The predicted accuracy rate of the Gaussian kernel SVM classifiers was almost identical to that obtained with the linear kernel SVM models (see Supporting Information S1 for details).

### Statistical evaluation of the ss-motif information content

In order to estimate to what extent the information content of the ss-motifs deviated from that randomly generated sequences, we randomly selected a set of regions (RSSs) from the pre-miRNA (positive) and CDS hairpin (negative) training sets with length distributions and nucleotide and structural notations corresponding to those in the actual ss-motifs generated from each respective training set. This procedure was repeated 10,000 times to estimate the probability (p-value) of the observed various characteristics in the actual ss-motif sets (see Supporting Information S1 for details).

### Evaluation of ss-motif similarity within miRNA families

In order to estimate the extent of ss-motif similarity within miRNA families, we analyzed three miRNA families recorded the number of ss-motifs common to all members of the family (see Table S5). This number was compared to the number of ss-motifs common to the same number of randomly selected pre-miRNAs, a procedure repeated 1000 times to estimate the statistical significance (p-value) of observing the number of common ss-motifs recorded for each family.

In a further effort to test for the effects of within-family similarity, we removed all (but one) of pre-miRNAs with sequence identity higher than 80%/70%, and repeated the entire procedure as given above, with the following modifications: The pre-miRNA set was reduced to 577/557 sequences, and a corresponding negative set was collected. The training and test sets were reduced to 462/442 and 115/112 sequences, respectively, and the Tereisias K parameter was changed to K = 433/413. Altogether 28941/23507 non-redundant ss-motifs were obtained, ranked, and introduced to a linear kernel SVM model in increments of 100, starting at N = 600 and ending at N = 1400.

### The positions of the ss-motifs

To estimate the positional distribution of each ss-motif, the pre-miRNA sequences were centered on the 5′ end (mir_start) of the mature miRNA (or miRNA*, whichever apply in each case), and the pre-miRNA length were normalized as given in equation (1) and Figure S3 (“mir_end” indicating the 3′ end of the miRNA* (or miRNA)).

The normalized position (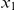
′) of an ss-motif was calculated as follows,
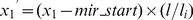
(1)

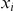
 indicating the actual position of the 5′end nucleotide of the ss-motif, 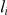 indicating the (mir_start – mir_end) difference for the pre-miRNA in question, and 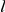 indicating the average (mir_start – mir_end) difference for all 608 pre-miRNA sequences.

### Software availability

The python program for the method is available for downloading at http://www.regulatoryrna.org/pub/mirident/index.html.

## Supporting Information

Supporting Information S1 Supplementary methods and results.
(DOC)

Figure S1 **Specific combinations of nucleotide and structural information.** A. Frequency of co-occurring nucleotide and structural notations. B. Three significantly enriched “neighbouring” nucleotide-structure notations among the pre-miRNA ss-motifs.
(TIF)

Figure S2 **Normalisation of ss-motif positions in a pre-miRNA sequence.** x1–x4 indicate ss-motif positions. Red sections indicate the positions of the mature miRNA/miRNA* sequences.
(TIF)

Figure S3 **Distribution of nucleotide notations.** Light hues (pink, light green) indicates the positive and negative randomly selected sequences (RSS). Darker hues (red, green) indicates the actual ss-motifs derived from the positive (pre-miRNA) and negative (CDS hairpin) training sets.
(TIF)

Table S1 List of all ss-motifs.
(XLS)

Table S2 SVM pre-miRNA prediction with increasing number (N) of features.
(DOC)

Table S3 SVM pre-miRNA prediction after adjusting for sequence similarity. The table shows prediction accuracy (ACC) after filtering out pre-miRNAs with sequences identity >70% or 80%, respectively. N denotes the number of feature (i.e., ss-motifs) included in the SVM.
(DOC)

Table S4 Statistical evaluation of the ss-motif information content.
(XLS)

Table S5 Three miRNA families used for ss-motif similarity analysis.
(DOC)
