# Supplementary material for: Integrated Sequence-Structure Motifs Suffice to Identify microRNA Precursors
Source: PLoS One. 2012 Mar 15;7(3):e32797. doi: 10.1371/journal.pone.0032797 (PMC3305290; doi:10.1371/journal.pone.0032797)
Supplement: Supporting Information S1 — Supplementary methods and results. (DOC) [file pone.0032797.s001.doc]

# Additional file

**Title: Supplementary methods and results.**

**Content:**

**Supplementary Methods**

**Supplementary Results**

# Supplementary Methods

## Data preprocessing

**Human pre-miRNA preprocessing**. We downloaded miRNA precursor from mirBase Version 11.0 . To avoid redundancies we applied a procedure from Rigoutsos *et al.* to remove sequences with sequence identity above 90 %, leaving 638 miRNA precursor sequences. These were subsequently subjected to secondary structure prediction using RNAfold , and structures with more than one predicted loop were removed, leaving a final set of 608 miRNA precursor sequences for further study.

**Human non-pre-miRNA preprocessing.** The human non-pre-miRNA data set was generated according to the following criteria:

(1) Sequence length between 51 and 137 nucleotides.

(2) The predicted secondary structure (RNAfold) should contain a stem of 18 or more paired bases (including GU pairs).

(3) The secondary structure should have a minimum free energy (MFE) of -15 kcal/mol or lower.

(4) The predicted secondary structure should not include more than one loop.

(5) No two sequences in the set should have sequence identity above 90 %.

Altogether 7879 sequences were extracted from human CDSs according to the above criteria, and a set of 608 sequences were randomly selected from these to constitute the human non-pre-miRNA sequence set.

## SVM for training and prediction

Due to its reported good generalization ability , a Support Vector Machine (SVM) procedure was adopted to classify pre-miRNAs versus non-pre-miRNA hairpins using the N-dimensional feature vectors as input (see Table S2 for details). First, the N features (the 968 sequences in the training sets) were rescaled linearly by the SVM-scale program to the interval [-1, 1 to avoid attributes in higher numeric ranges dominating those in smaller numeric ranges, as well as to avoid other numerical difficulties during the calculation. Each classifier was trained with the ‘SVM-train’ linear kernel, and thereafter the classification was conducted on the testing and independent evaluation data sets with ‘svm-predict’. After sorting the motifs by “$w$” (the weight of the ss-motifs) obtained with the linear kernel SVM model, we sequentially introduced batches of 100 ss-motifs from the top of the sorting list (*i.e*, the first 100, 200, and so on) until the first 1500 ss-motifs had been used. Thereafter, the first 2000, 2500, 5000, 10000, 20000 ss-motifs, and, at last, all the 29734 ss-motifs were used. As seen from Figure 2A, the prediction accuracy rate by the classifiers increase with small fluctuations and reach optimal performance (ACC=98.39%) at 1300 ss-motifs, where after the accuracy decreases with increasing number of ss-motifs.

To test the validity of the result obtained with linear kernel SVM, we also trained the classifiers using Gaussian kernel SVM with N (N=100, 200…) ss-motifs as described above. All classifier models were generated with ‘svm-train’$-C 2^{C} -g \gamma$, and default RBF kernel. As both the penalty parameter$C$and the RBF kernel parameter$\gamma$are critical for the SVM performance , they were optimally calibrated by an exhaustive grid-search strategy, which was carried using ‘grid.py’ as described by Ng and Mishra . In short, for each hyper parameter pair $(C,\gamma)$selected from the search space $\log_{2}C\in[-5,-3,-1,\cdots,13,15]$ and$\log_{2}\gamma\in[-15,-13,-11,\cdots,1,3]$, we performed a 5-fold cross validation. The training data set was randomly partitioned into approximately five distinct equal-sized subsets. Repeating the validation process five times for each subset *i.e.* retaining one set for testing and using the remaining four sets for training, the average accuracy of the five models produced a 5-fold leave-one-out cross-validation (LOOCV) accuracy rate . To avoid over-fitting the generalization, the combination of hyper parameters $(C,\gamma)$ maximizing the 5-fold LOOCV accuracy rate served as the default setting for training classifier. Finally, classification was conducted on the testing and independent evaluation data sets with ‘svm-predict’. The predicted accuracy rate of the Gaussian kernel SVM classifiers was almost identical to that obtained with the linear kernel SVM models.

## Statistical evaluation of the ss-motif information content

In order to estimate to what extent the information content of the ss-motifs deviated from that randomly generated sequences, we randomly selected a set of sequences (RSSs) from the pre-miRNA (positive) and CDS hairpin (negative) training sets with length distributions corresponding to those of the 941 and 535 ss-motifs derived from the two respective training sets. Next, for each of these randomly selected sequences, we randomly removed a number nucleotide and structural notations (that is, the specific nucleotides and structural notations were replaced with respectively “N” and “S”), so that the numbers of remaining nucleotides and structural notations corresponded to those in the actual ss-motifs generated from each respective training set (see Table S4) This procedure was repeated 10000 times, thereby generating 10000 sets of randomly generated sequences for each training set. Assuming normal distribution of the nucleotide and structural “notations” in the randomly generated sequenced, their distributions were used to estimate the probability (p-value) of the observed various characteristics in the actual ss-motif sets.

# Supplementary Results

## The ss-motifs are biased towards structural information content

The 1300 ss-motifs used for pre-miRNA prediction included an average sequence and structure information from 6.3 nucleotides (median= 6 nt, min.= 2 nt, max.= 15 nt). Of the 1300 ss-motifs, 941 were extracted from the positive (pre-miRNA) training set, and 553 from the negative set (194 ss-motifs having been extracted from both training sets, see Supplementary Table S1). The overall information content in terms of specific nucleotide (A, U, C, G) and structure (L, R, D) notations in these 1300 ss-motifs included 6431 specific notations (“N” and “S” excluded), with a substantial bias towards structural information (72.9% of all notations), 206 ss-motifs being exclusively composed of structural notations (Supplementary Table S1). No motifs spanned the loop (*i.e.*, contained both left and right intra-molecular notations), whereas 144 ss-motifs contained only specific notations for absence (*i.e.,* “D”) of intra-molecular interactions (Supplementary Table S1), the latter type of ss-motifs predominantly deriving from the negative training set. The structural notations were nearly equally distributed between “L” (36.6 %) and “R” (40.3 %), while 23.0 % (Supplementary Table S4) of the structural notations specifically indicated absence of intra-molecular interactions (*i.e.*, “D”).

We carried out a separate analysis of the nucleotide and structural notation content in ss-motifs derived from the positive (pre-miRNA) and negative (CDS hairpin) training sets. For comparison, we simulated a random selection of sequences (RSSs) from the two training sets (region lengths and overall number of nucleotide and structural notations being identical to those of the real ss-motifs), and repeated the simulation 10,000 times (see Materials and Methods). The relative distribution of specific notations in the RSS sets necessarily closely reflected those of the respective training sets.

The distribution of nucleotide and structural notation of the ss-motifs derived from the positive and negative sets differed markedly between themselves and from their respective sets of RSSs. The structural notations of the pre-miRNA ss-motifs were significantly enriched for both left (“L”) and right (“R”) (p=3.51×10^-6^ and p=7.81×10^-11^, respectively) notations, and strongly depleted of “D” notations (p= 1.27×10^-63^) compared to the RSSs (Figure 3B). The CDS hairpin ss-motifs were, on the other hand, significantly depleted in left notations (p=2.92×10^-5^) and had much higher numbers of specific “D” notations (p=2.73×10^-7^) than had the RSSs. Thus, although the number of intra-molecular interactions was higher in the positive than in the negative training set (Figure 3B), these differences were accentuated in the two ss-motifs sets, and particularly so with respect to the usage of information on absence of intra-molecular interactions.

## The ss-motif nucleotide contents deviate from those of the training sets

The nucleotide composition of the two training sets differed to some extent, the positive (pre-miRNA) training set having a higher content of U residues and lower contents of G and C residues than the negative training set (CDS hairpins; Figure S1 ). The nucleotide notations of the two ss-motif sets, however, do not slavishly reflect these differences. In the pre-miRNA ss-motifs, the number of U notations is greatly enriched (p<10^-100^) above the (already high) content in the RSS set, whereas the number of C and A notations are greatly reduced (p<3×10^-12^). Though it has previously been noted that pre-miRNA sequences have a higher AU content than non-pre-miRNA hairpins , the data suggests that U residues play a considerably larger role than A residues in defining a pre-miRNA. Similarly, it can be inferred that even though C residues are tolerated in pre-miRNA sequences, they contribute very little to the recognition of the miRNA precursors by the miRNA processing apparatus. The observation that C notations are not enriched in the CDS hairpins supports this.

In the CDS hairpin ss-motifs, G notations are significantly enriched (p=1.48×10^-12^) and U notations significantly (p=3.57×10^-5^) depleted. The absence of U residues appears logical in the light of their apparent high information value in defining miRNA precursors. G residues are relatively common in both training sets, however, the relative enrichment of G notations in CDS hairpin ss-motifs is not mirrored by a similar depletion in pre-miRNA ss-motifs, thus, the somewhat illogical situation arises in which the presence of G residues in non-pre-miRNA sequences appears to contribute substantially to their rejection by the miRNA processing apparatus, whereas at the same time G residues in pre-miRNA sequences apparently do not have this effect. The explanation may lie in the structural context in which G residues reside. ss-motifs without positive notations on intra-molecular interactions (*i.e.*, without “L” and “R” notations, see Table S1) are far more abundant among the CDS hairpin ss-motifs, and in this category of ss-motifs, notations of G are far more frequent than other nucleotide notations. It may thus be that G residues in a context of less pronounced intra-molecular interactions (*e.g.*, in the loop) is a strong signal for rejection by the miRNA processing apparatus.

We next analysed to which extent specific nucleotide and structural notations tended to co-occur in the ss-motifs. Overall, significantly fewer positions in both the pre-miRNA and CDS hairpin ss-motifs contained combined nucleotide and structural notations (*e.g.*, A_L_) than in the respective RSS sets (p=4.20×10^-52^ and p=2.11×10^-23^, respectively; Figure 3A). We therefore asked whether the sequence and structure information was combined in other forms, for example by nearest neighbor combinations of nucleotide and intra-molecular notations. However, both combinations of nucleotide notations with specific structural notation for the immediate upstream nucleotide (*e.g.*, N_L_A_S_) or downstream nucleotide (*e.g.*, A_S_N_L_) were less frequent in both sets of ss-motifs (p<5×10^-4^) than in the respective RSS sets (Figure 3A). There was nonetheless a notable difference between the two ss-motif sets with respect to specific combinations of nucleotide and structural information within or among neighboring positions. In ss-motifs derived from the CDS hairpins the frequencies of specific nucleotide-structure notations did generally not deviate from those observed in the randomly selected motifs (at significance level p = 10^-3^; supplementary Table S4). The two sole exceptions were the notations “A_L_” and “A_R_”, which were significantly depleted (p <6.5×10^-4^) in both ss-motif sets (Supplementary Figure S2 A). In contrast, in the pre-miRNA ss-motifs, more than a half of all possible nucleotide-structure notations within or among neighboring position deviated significantly from the frequencies observed in the randomly selected motifs (Supplementary Table S4). As expected from the generally reduced frequency of such combinations (see Fig. 3C in the main text), nearly all these combinatorial notations were depleted relative to the randomly selected motifs. The three cases of nucleotide-structure combinations that were significantly (p < 8.02×10^-4^) enriched in the pre-miRNA ss-motifs all included nucleotide notations combined with structural notations for the nearest upstream position (Supplementary Figure S2, B, above). Notably, these included the “N_L_G_S_” combination, further supporting the idea that informational content of G residues depends strongly on its structural context, since G notations are relatively abundant in ss-motifs derived from both the positive and negative training set (see above). The two other significantly enriched combinatorial notations both included U notations combined positive structural for the nearest upstream neighboring position (i.e., “N_L_U_S_”, and “N_R_U_S_”, Supplementary Figure S2, B). These two combinatorial notations are also the most abundant in the entire pre-miRNA ss-motif set, exceeding the number of U_L_ and U_R_ combinations by 34% and 40 %, respectively (Supplementary Table S4). It is also peculiar that it is the structural notation of the upstream neighbor that is the most important context for a U residue, irrespective of whether the residue occurs on the left or right arm of the RNA stem. While U notations are the most enriched nucleotide notations in the pre-miRNA ss-motifs, it is also apparent that the informational and/or functional role of the U residues can depend on very specific structural contexts

# References

## 1. Kozomara, A. and S. Griffiths-Jones, miRBase: integrating microRNA annotation and deep-sequencing data. Nucleic Acids Res, 2011. 39(Database issue): p. D152-7.

## 2. Rigoutsos, I., *et al*., Short blocks from the noncoding parts of the human genome have instances within nearly all known genes and relate to biological processes. Proc Natl Acad Sci U S A, 2006. 103(17): p. 6605-10.

## 3. Hofacker, I.L., Vienna RNA secondary structure server. Nucleic Acids Res, 2003. 31(13): p. 3429-31.

## 4. Cherkassky, V., The nature of statistical learning theory. IEEE Trans Neural Netw, 1997. 8(6): p. 1564.

## 5. Duan, K.e.a., Evaluation of simple performance measures for tuning SVM hyperparameters. Neurocomputing, 2003. 51: p. 41–59.

## 6. Ng, K.L. and S.K. Mishra, De novo SVM classification of precursor microRNAs from genomic pseudo hairpins using global and intrinsic folding measures. Bioinformatics, 2007. 23(11): p. 1321-30.

## 7. Batuwita, R. and V. Palade, microPred: effective classification of pre-miRNAs for human miRNA gene prediction. Bioinformatics, 2009. 25(8): p. 989-95.
