# Supplementary figures and images for: Integrated Sequence-Structure Motifs Suffice to Identify microRNA Precursors
Source: PLoS One. 2012 Mar 15;7(3):e32797. doi: 10.1371/journal.pone.0032797 (PMC3305290; doi:10.1371/journal.pone.0032797)

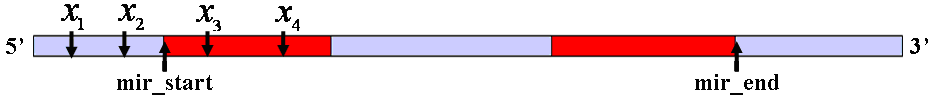

Supplement: Figure S1 — Specific combinations of nucleotide and structural information. A. Frequency of co-occurring nucleotide and structural notations. B. Three significantly enriched “neighbouring” nucleotide-structure notations among the pre-miRNA ss-motifs. (TIF) [file pone.0032797.s002.tif]

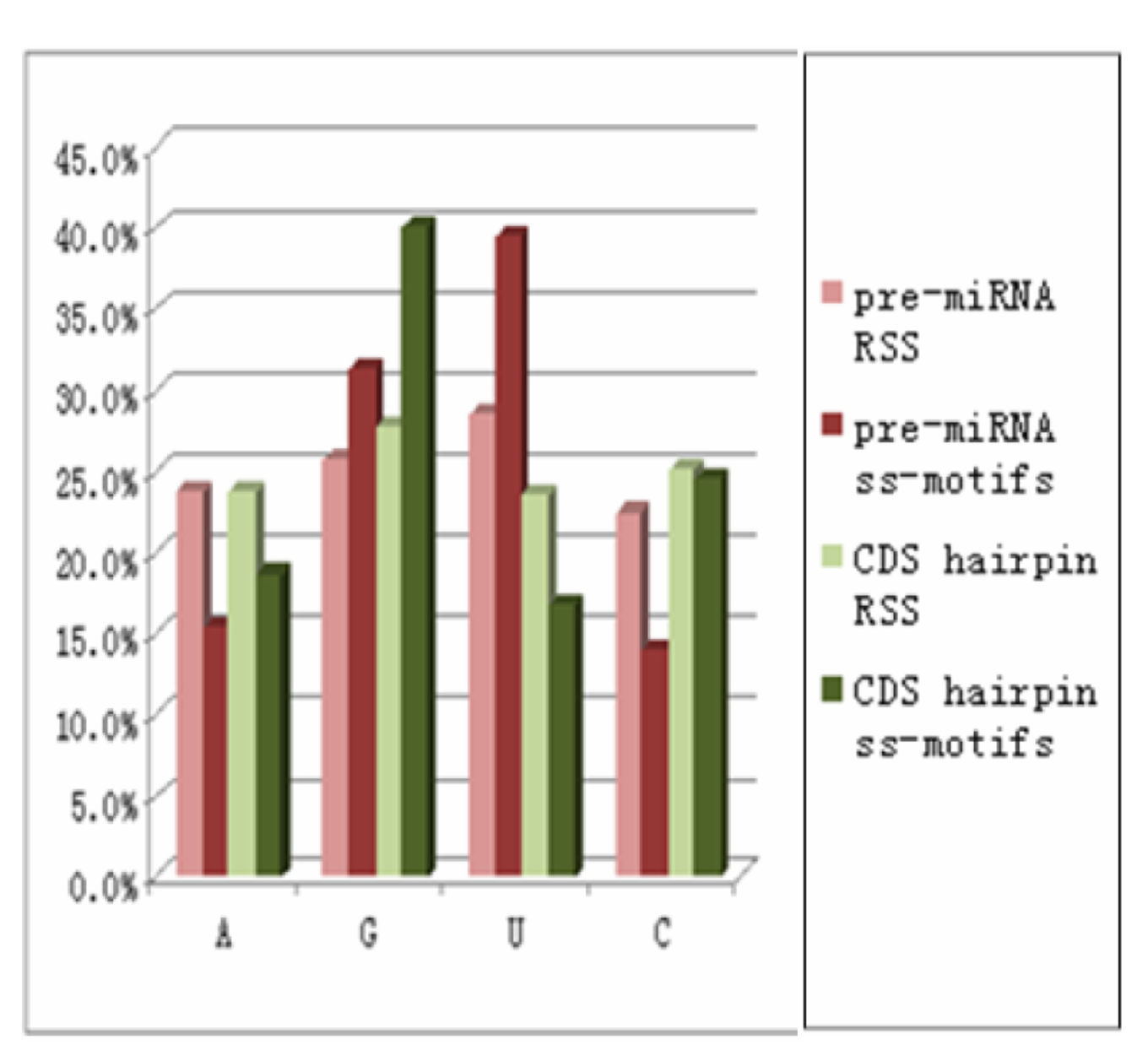

Supplement: Figure S2 — Normalisation of ss-motif positions in a pre-miRNA sequence. x1–x4 indicate ss-motif positions. Red sections indicate the positions of the mature miRNA/miRNA* sequences. (TIF) [file pone.0032797.s003.tif]

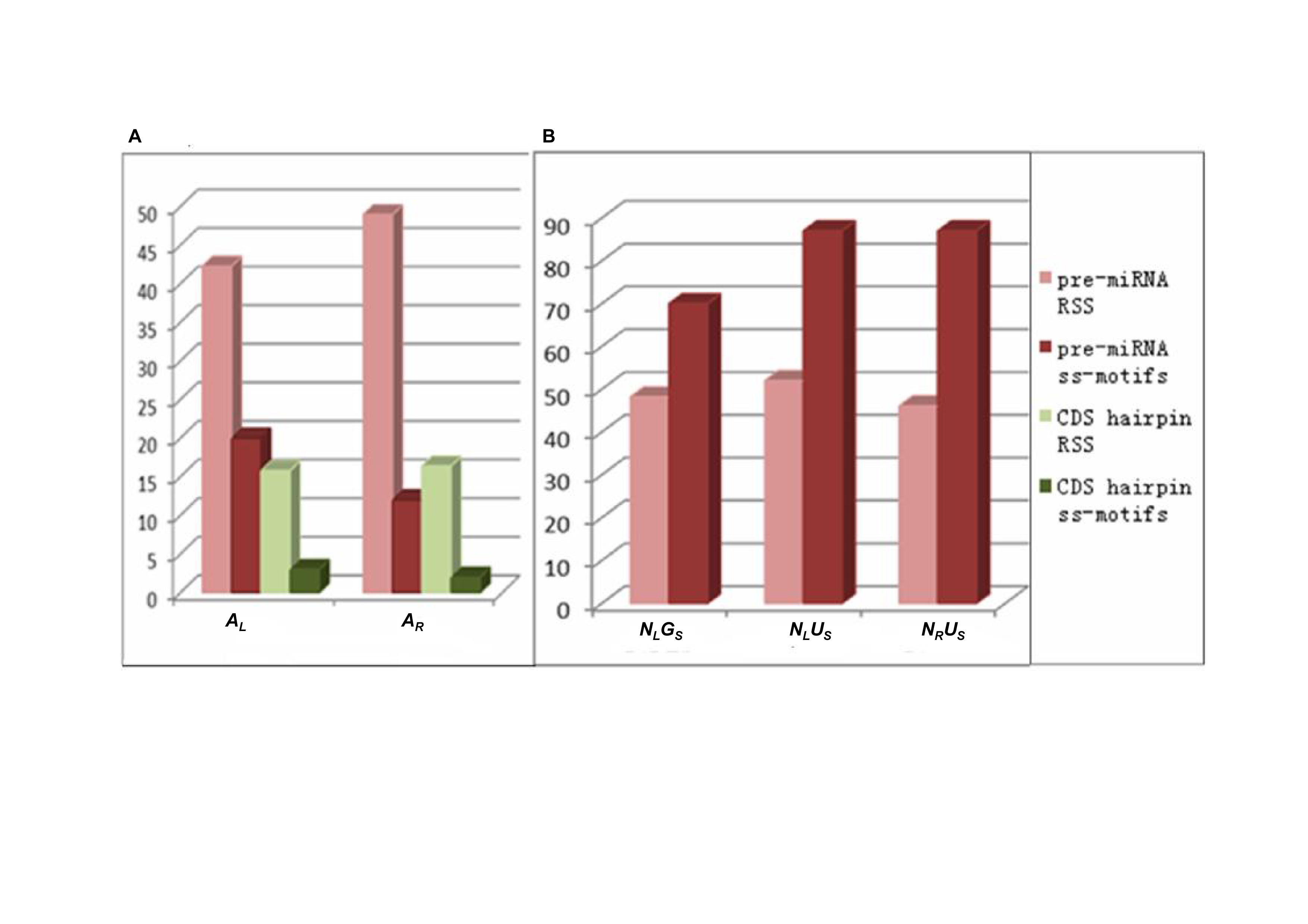

Supplement: Figure S3 — Distribution of nucleotide notations. Light hues (pink, light green) indicates the positive and negative randomly selected sequences (RSS). Darker hues (red, green) indicates the actual ss-motifs derived from the positive (pre-miRNA) and negative (CDS hairpin) training sets. (TIF) [file pone.0032797.s004.tif]
