# Supplementary material for: Integrated Sequence-Structure Motifs Suffice to Identify microRNA Precursors
Source: PLoS One. 2012 Mar 15;7(3):e32797. doi: 10.1371/journal.pone.0032797 (PMC3305290; doi:10.1371/journal.pone.0032797)
Supplement: Table S2 — SVM pre-miRNA prediction with increasing number (N) of features. (DOC) [file pone.0032797.s006.doc]

## Table S2 . SVM pre-miRNA prediction with increasing number (N) of features.

| N | Training ACC (%) | c | g | Testing ACC (%)  (Gauss kernel) | Testing ACC (%)  (linear kernel) |
| --- | --- | --- | --- | --- | --- |
| 100 | 91.219 | 512 | 0.00012207 | 90.3226 | 91.129 |
| 200 | 91.6322 | 8 | 0.0078125 | 93.1452 | 91.129 |
| 300 | 93.2851 | 2 | 0.03125 | 93.1452 | 93.5485 |
| 400 | 93.905 | 8 | 0.0078125 | 94.7581 | 94.7581 |
| 500 | 94.8347 | 128 | 0.00012207 | 93.9516 | 96.371 |
| 600 | 95.0413 | 512 | 0.000488281 | 94.7581 | 94.3548 |
| 700 | 95.9711 | 128 | 0.00012207 | 95.1613 | 94.7581 |
| 800 | 96.3843 | 512 | 0.00012207 | 95.1613 | 95.5645 |
| 900 | 95.9711 | 32 | 0.000488281 | 95.9677 | 97.1774 |
| 1000 | 96.281 | 2048 | 0.0000305176 | 95.9677 | 95.9677 |
| 1100 | 96.4876 | 2048 | 0.0000305176 | 97.5806 | 97.5806 |
| 1200 | 96.3843 | 512 | 0.00012207 | 97.9839 | 97.5806 |
| 1300 | **96.4876** | 128 | 0.000488281 | **97.9839** | **98.39** |
| 1400 | 96.5909 | 512 | 0.00012207 | 96.371 | 97.5806 |
| 1500 | 96.3843 | 2048 | 0.0000305126 | 97.9839 | 97.9839 |
| 2000 | 96.281 | 128 | 0.00012207 | 97.5806 | 97.5806 |
| 2500 | 96.1777 | 128 | 0.00012207 | 97.5806 | 97.5806 |
| 5000 | 95.1446 | 128 | 0.0000305176 | 96.371 | 96.7742 |
| 10000 | 92.5625 | 128 | 0.00012207 | 93.9516 | 94.3548 |
| 20000 | 90.3926 | 8 | 0.00012207 | 91.5323 | 91.129 |
| 29734 | 89.876 | 2 | 0.00012207 | 87.5 | 87.0968 |

Training ACC is the max 5-fold cross-validation accuracy rate on the optimal hyper-parameters c and g on the training data set. Testing ACC (Gaussian kernel/ linear kernel) is the predicted accuracy rate conducting the Gaussian kernel/linear kernel SVM model on the test set.
