# Supplementary material for: Integrated Sequence-Structure Motifs Suffice to Identify microRNA Precursors
Source: PLoS One. 2012 Mar 15;7(3):e32797. doi: 10.1371/journal.pone.0032797 (PMC3305290; doi:10.1371/journal.pone.0032797)
Supplement: Table S3 — SVM pre-miRNA prediction after adjusting for sequence similarity. The table shows prediction accuracy (ACC) after filtering out pre-miRNAs with sequences identity >70% or 80%, respectively. N denotes the number of feature (i.e., ss-motifs) included in the SVM. (DOC) [file pone.0032797.s007.doc]

## Table S3. SVM pre-miRNA prediction after adjusting for sequence similarity. The table shows prediction accuracy (ACC) after filtering out pre-miRNAs with sequences identity > 70% or 80%, respectively. N denotes the number of feature (i.e., ss-motifs) included in the SVM.

|  | ACC (%) | |
| --- | --- | --- |
| N | Sequence identity < 70% | Sequence identity  < 80% |
| 600 | 96.4 | 94.3 |
| 700 | 93.9 | 93.9 |
| 800 | 96.9 | 95.7 |
| 900 | 96.9 | 96.1 |
| 1000 | 96.9 | 96.1 |
| 1100 | 96.4 | 96.1 |
| 1200 | 96.4 | 95.7 |
| 1300 | 96.4 | 97.4 |
| 1400 | 96.9 | 97.4 |
