# Supplementary material for: Integrated Sequence-Structure Motifs Suffice to Identify microRNA Precursors
Source: PLoS One. 2012 Mar 15;7(3):e32797. doi: 10.1371/journal.pone.0032797 (PMC3305290; doi:10.1371/journal.pone.0032797)
Supplement: Table S5 — Three miRNA families used for ss-motif similarity analysis. (DOC) [file pone.0032797.s009.doc]

## Table S5. Three miRNA families used for ss-motif similarity analysis.

| miRNA gene family mir-515 | miRNA gene  Family mir-154 | miRNA gene  family let-7 |
| --- | --- | --- |
| hsa-mir-520e | hsa-mir-154 | hsa-let-7a-2 |
| hsa-mir-515-1 | hsa-mir-369 | hsa-let-7a-3 |
| hsa-mir-519e | hsa-mir-377 | hsa-let-7c |
| hsa-mir-520f | hsa-mir-381 | hsa-let-7e |
| hsa-mir-520a | hsa-mir-382 | hsa-let-7f-1 |
| hsa-mir-526b | hsa-mir-323 | hsa-mir-98 |
| hsa-mir-525 | hsa-mir-409 | hsa-let-7g |
| hsa-mir-523 | hsa-mir-410 | hsa-let-7i |
| hsa-mir-518b | hsa-mir-487a |  |
| hsa-mir-526a-1 | hsa-mir-494 |  |
| hsa-mir-520c | hsa-mir-496 |  |
| hsa-mir-518c | hsa-mir-539 |  |
| hsa-mir-524 | hsa-mir-487b |  |
| hsa-mir-519d | hsa-mir-655 |  |
| hsa-mir-520d | hsa-mir-656 |  |
| hsa-mir-520g | hsa-mir-1185-1 |  |
| hsa-mir-516b-2 | hsa-mir-300 |  |
| hsa-mir-526a-2 |  | |
| hsa-mir-518e |  | |
| hsa-mir-518d |  | |
| hsa-mir-516b-1 |  | |
| hsa-mir-517c |  | |
| hsa-mir-520h |  | |
| hsa-mir-521-1 |  | |
| hsa-mir-519a-2 |  | |
| hsa-mir-1283-1 |  | |

The lists do not include pre-miRNAs excluded by the >90% sequence identity criterion (see Supplementary methods)
